# Supplementary material for: The interactome of intact mitochondria by cross-linking mass spectrometry provides evidence for coexisting respiratory supercomplexes
Source: Mol Cell Proteomics. 2017 Dec 8;17(2):216–32. doi: 10.1074/mcp.RA117.000470 (PMC5795388; doi:10.1074/mcp.RA117.000470)
Supplement: Supplemental Data [file supp_RA117.000470_133922_0_supp_23978_fzffwf.zip › spectra_annotation/mito_DR_spectra_annotation/299-1-4-1-4-1.pdf]

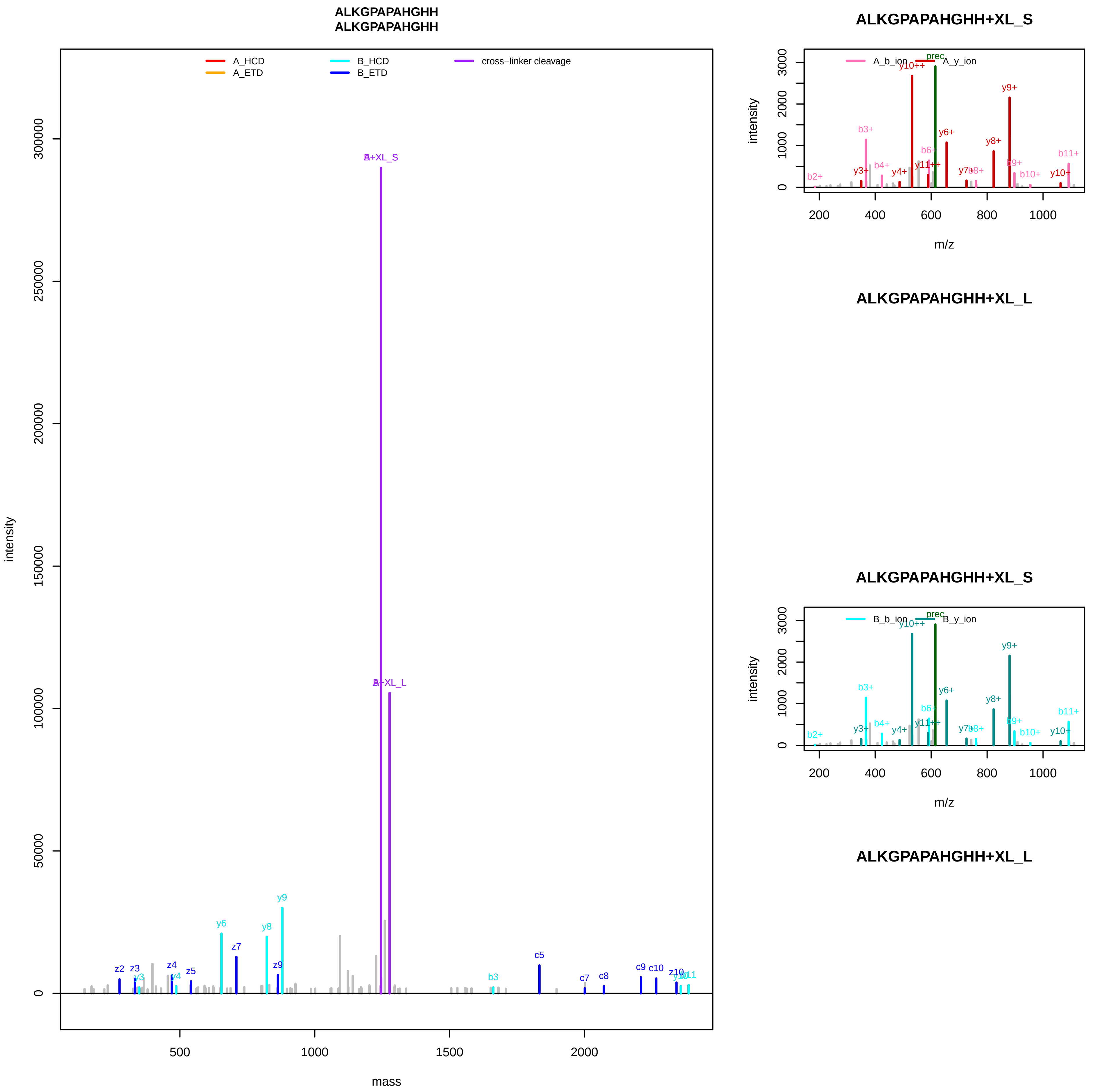

ALKGPAPAHGHH
ALKGPAPAHGHH
A_HCD
A_ETD
B_HCD
B_ETD
cross−linker cleavage
B+XL_S
B+XL_L
y6
y8
y9
z2
z3
y3
z4
y4
z5
z7
z9
b3
c5
c7
c8
c9
c10
z10
y10
y11
intensity
mass
0
50000
100000
150000
200000
250000
300000
500
1000
1500
2000
ALKGPAPAHGHH+XL_S
A_b_ion
A_y_ion
prec
y10++
y9+
b3+
y6+
b6+
y8+
b4+
y4+
y11++
y3+
b2+
y7+
b8+
b9+
b10+
y10+
b11+
intensity
m/z
0
1000
2000
3000
200
400
600
800
1000
ALKGPAPAHGHH+XL_L
ALKGPAPAHGHH+XL_S
B_b_ion
B_y_ion
prec
y10++
y9+
b3+
y6+
b6+
y8+
b4+
y4+
y11++
y3+
b2+
y7+
b8+
b9+
b10+
y10+
b11+
intensity
m/z
0
1000
2000
3000
200
400
600
800
1000
ALKGPAPAHGHH+XL_L
